# The Involvement of SMILE/TMTC3 in Endoplasmic Reticulum Stress Response

**DOI:** 10.1371/journal.pone.0019321

**Published:** 2011-05-16

**Authors:** Maud Racapé, Jean-Paul Duong Van Huyen, Richard Danger, Magali Giral, Françoise Bleicher, Yohann Foucher, Annaïck Pallier, Paul Pilet, Petra Tafelmeyer, Joanna Ashton-Chess, Emilie Dugast, Ségolène Pettré, Béatrice Charreau, Jean-Paul Soulillou, Sophie Brouard

**Affiliations:** 1 Institut National de la Santé Et de la Recherche Médicale Unité Mixte de Recherche 643 and Institut de Transplantation Urologie-Néphrologie, Nantes, France; 2 Université de Nantes, Nantes, France; 3 Centre Hospitalier Universitaire Hôtel-Dieu, Nantes, France; 4 Institut National de la Santé Et de la Recherche Médicale U970, PARCC, Hôpital Européen Georges Pompidou, Université Paris Descartes, Paris, France; 5 Université de Lyon, Université Lyon 1, Institut de Génomique Fonctionnelle de Lyon, Unité Mixte de Recherche 5242 et Centre National de la Recherche Scientifique, Ecole Normale Supérieure de Lyon, Lyon, France; 6 Faculté d'Odontologie, Institut National de la Santé Et de la Recherche Médicale EMI 9903, Nantes, France; 7 Hybrigenics Services SAS, Paris, France; Universidade de Sao Paulo, Brazil

## Abstract

**Background:**

Thestate of operational tolerance has been detected sporadically in some renal transplanted patients that stopped immunosuppressive drugs, demonstrating that allograft tolerance might exist in humans. Several years ago, a study by Brouard *et al.* identified a molecular signature of several genes that were significantly differentially expressed in the blood of such patients compared with patients with other clinical situations. The aim of the present study is to analyze the role of one of these molecules over-expressed in the blood of operationally tolerant patients, SMILE or TMTC3, a protein whose function is still unknown.

**Methodology/Principal Findings:**

We first confirmed that SMILE mRNA is differentially expressed in the blood of operationally tolerant patients with drug-free long term graft function compared to stable and rejecting patients. Using a yeast two-hybrid approach and a colocalization study by confocal microscopy we furthermore report an interaction of SMILE with PDIA3, a molecule resident in the endoplasmic reticulum (ER). In accordance with this observation, SMILE silencing in HeLa cells correlated with the modulation of several transcripts involved in proteolysis and a decrease in proteasome activity. Finally, SMILE silencing increased HeLa cell sensitivity to the proteasome inhibitor Bortezomib, a drug that induces ER stress *via* protein overload, and increased transcript expression of a stress response protein, XBP-1, in HeLa cells and keratinocytes.

**Conclusion/Significance:**

In this study we showed that SMILE is involved in the endoplasmic reticulum stress response, by modulating proteasome activity and XBP-1 transcript expression. This function of SMILE may influence immune cell behavior in the context of transplantation, and the analysis of endoplasmic reticulum stress in transplantation may reveal new pathways of regulation in long-term graft acceptance thereby increasing our understanding of tolerance.

## Introduction

The routine monitoring of renal allograft survival in humans depends on functional clinical parameters such as blood creatinine clearance, proteinuria level, the presence of circulating anti-HLA and donor specific antibodies and scoring of intra-graft lesions in graft biopsies. Standard immunosuppressive drugs are non-specific, increase opportunistic infections and malignancies and can be nephrotoxic [1]. Immune tolerance, which has been achieved in several experimental models [2], might provide a means of avoiding such inherent problems since immunosuppressive treatment could be reduced or completely withdrawn in tolerant patients. Although this phenomenon (induced or “spontaneous”) is rare in renal transplantation in primates and humans, several studies have shown its clinical feasibility [3], [4], [5]. Identifying and understanding the biological features characterizing operational tolerance may unveil molecular mechanisms allowing such patients to tolerate their graft without immunosuppression treatment. We previously identified 49 genes differentially expressed in the blood of operationally tolerant patients compared to stable patients under classical immunosuppressive therapy, patients with chronic antibody-mediated rejection and healthy volunteers [6]. These genes were shown to be able to correctly classify most of the patients according to their clinical status. Among these genes, we focused on SMILE, also called TMTC3 (transmembrane and tetratricopeptide repeat containing 3 protein), because it was one of the 13 genes that were over-expressed in the blood of operationally tolerant patients and because its function was still unknown. SMILE is a 7203 bp mRNA (NM_181783) and a 914 amino acid transmembrane protein (NP_861448). The protein presents the particularity of 10 tetratricopeptide repeats (TPRs, according to the UniProtKB website, http://www.uniprot.org/uniprot/Q6ZXV5), a pattern ubiquitously conserved through evolution and species. TPR-containing proteins are involved in several cellular functions such as molecular chaperone complexes, anaphase promoting complexes, transcription repression complexes, protein import complexes and protein folding [7]. They are found in a variety of different organisms and in various sub-cellular locations such as the cytosol, nucleus, mitochondria and peroxisomes [7]. The involvement of these motifs and the importance of their interactions for molecular and cellular functions have thus been shown in a number of different biological systems [7].

The aim of our study was to analyse the cellular and molecular function of SMILE/TMTC3 *in vitro* and the global pathways in which it is involved. In this study we report that SMILE interacts with PDIA3, a molecule involved in protein folding, and is involved in response to endoplasmic reticulum (ER) stress, which may play a role in immune regulation.

## Results

### SMILE transcripts are differentially expressed in PBMCs from operationally tolerant kidney transplant patients compared to stable patients and patients with chronic antibody-mediated rejection

**Figure 1:**
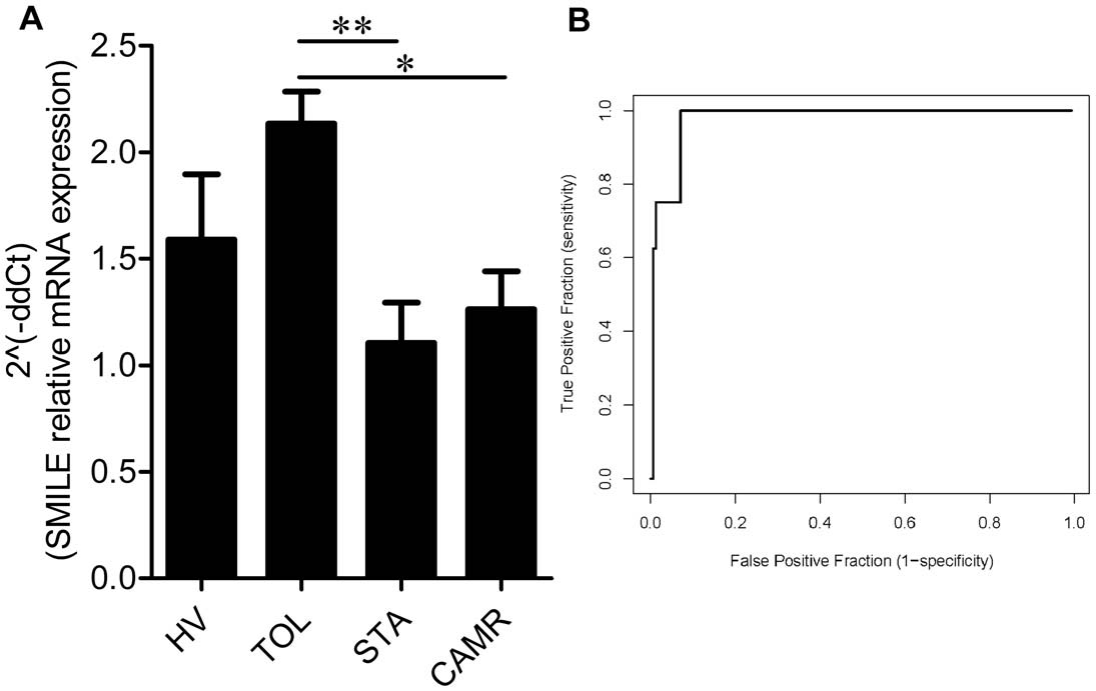
SMILE mRNA profile in renal transplant patients. (A) SMILE mRNA transcripts were increased in the PBMC of operationally tolerant patients (TOL, n = 8) compared to patients with stable graft function under standard immunosuppressive therapy (STA, n = 9, **p<0.01) and deteriorating graft function under standard immunosuppressive therapy with biopsy-proven chronic antibody-mediated rejection (CAMR, n = 14, *p<0.05) (*p = 0.0205, Kruskal-Wallis test,). (B) The ROC curve measuring the ability of SMILE mRNA quantity to correctly classify operationally tolerant patients versus patients with stable function.

In order to confirm the previous finding of SMILE mRNA differential expression in the blood of operationally tolerant patients compared to stable and chronic rejection patients by microarrays [6], SMILE mRNA levels were analyzed in the PBMCs of healthy volunteers (HV, n = 11), operationally tolerant patients (TOL, n = 8), and patients under standard immunosuppressive therapy with either stable graft function (STA, n = 9) or deteriorating graft function with biopsy-proven chronic antibody-mediated rejection (CAMR, n = 14). As shown in Figure 1A, SMILE mRNA was significantly differentially expressed in the PBMCs of TOL patients compared with STA (**p<0.01) and CAMR patients (*p<0.05) (Kruskal-Wallis test, *p* = 0.0205). The difference in transcript expression in the PBMCs of operationally tolerant patients was also confirmed compared to a larger cohort of patients with chronic rejection (19 patients) and a larger cohort of stable patients (164 patients) (Figure S1). The capacity of SMILE transcripts to distinguish between operationally tolerant patients and stable patients (Figure 1B) was studied by receiver operating characteristic (ROC) curve analysis. This analysis revealed a very good discriminative power for SMILE to distinguish TOL patients from STA patients with an optimal threshold of 1.23 (area under the curve [AUC] = 0.98; 95% confidence interval 0.95 to 1, good sensitivity of 1 and good specificity of 0.93). A ROC curve analysis also determined that the capacity of SMILE transcripts to distinguish between operationally tolerant patients and patients with chronic antibody-mediated rejection was also very good, with an optimal threshold of 1.86 (area under the curve [AUC] = 0.83; 95% confidence interval 0.66 to 0.96, good sensitivity of 0.77 and good specificity of 0.75) (Figure S2).

Furthermore, in a homogeneous cohort of 164 stable patients with a well characterized clinical status: stable renal function (STA) for more than five years under standard immunosuppressive therapy (thirty percent of these stable patients under Prograf and seventy percent under Cyclosporin A treatment), we showed that the level of SMILE mRNA was independent of quantitative variables, including time post-transplantation, creatinine clearance, proteinuria, HLA incompatibilities and recipient and donor age (Figure S3). Similarly, SMILE mRNA levels were also shown to be independent of qualitative variables (described as frequencies) such as recipient and donor gender, presence of anti-HLA antibodies or types of immunosuppressive treatment (Figure S4). Together, these results suggest that SMILE may be a good biomarker of transplant status.

### SMILE is involved in protein metabolism

**Figure 2:**
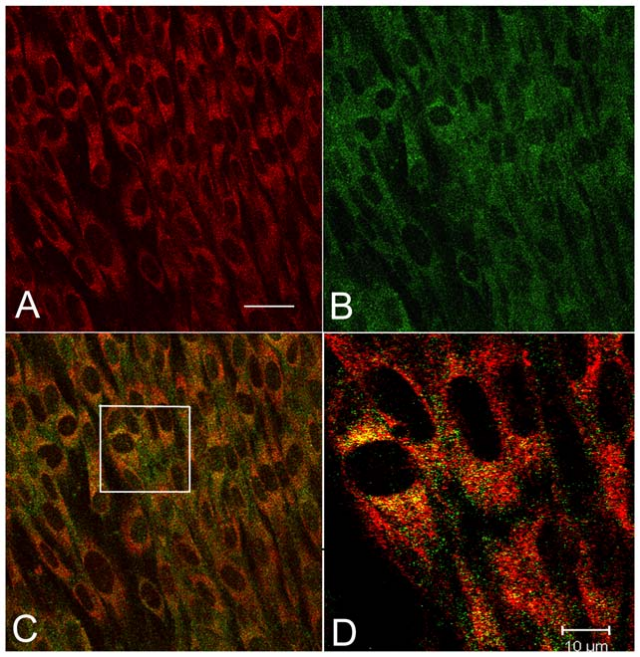
Confocal laser analysis of PDIA3 (red) and SMILE (green) proteins in cultured human odontoblasts. (A) PDIA3 labeling is localized in the endoplasmic reticulum. (B) SMILE labeling is mainly present in vesicles and in some cells in the reticulum area. (C) Merged picture showing the colocalization of PDIA3 and SMILE in the endoplasmic reticulum. (D) A higher magnification of (C) showing yellow dots in the endoplasmic reticulum. Bar in A is 40 µm. Bar in D is 10 µm.

SMILE was identified as a high confidence prey (Predicted Biological Score A [8]) in a yeast two hybrid screen with Protein Disulfide Isomerase family A member 3 (PDIA3 or GRP58) as bait, performed on a random-primed human brown adipocyte cDNA library (Figure S5). PDIA3 is involved in the folding of glycoproteins by disulfide bond formation in the ER and is over-expressed in ER stress [9]. Double-staining of SMILE and PDIA3 in odontoblast cultures (Figure 2C and D) also showed that SMILE and PDIA3 colocalized in the endoplasmic reticulum, confirming that these two molecules can interact in the ER.

To determine the role of SMILE in the cell, we studied SMILE transcript modulation in the HeLa cell line. SMILE mRNA expression was checked by RT-PCR and decreased by almost 84% in resting HeLa cells transfected with SMILE siRNA as compared to cells transfected with the Stealth RNAi negative control Low GC (Figure S6, ***p = 0.0002, Mann-Whitney test, mean replicate values of three independent experiments). High throughput microarray analysis was performed on resting HeLa cells transfected with SMILE or negative control siRNA in order to identify differentially expressed genes and to define cellular functions affected by SMILE silencing. Signals were studied with a SAM analysis (FDR = 0.0011, number of permutations: 5000). Overall, 549 and 532 genes were significantly up- and down-regulated respectively in cells transfected with SMILE siRNA as compared to cells transfected with negative control siRNA. Each list of up-regulated and down-regulated genes was analyzed using the GOminer website (http://discover.nci.nih.gov/gominer/) to define enrichment in several key biological functions. In this approach a function was defined by a GO number. One gene can have several GO numbers meaning that it can be involved in several mechanisms. We defined a set of 24 enriched functions for the list of down-regulated genes (Table 1). This classification was performed based on GO categories with enrichment *p-values*<0.05, and categories with at least 10 differentially expressed genes among the total genes involved in the function were selected. Among the down-regulated gene functions of SMILE siRNA-transfected cells, those concerning protein metabolic processes (GO:0019538 line 13 Table 1, GO:0044260 line 9 Table 1 and GO:0044267 line 16 Table 1) were particularly represented, such as catabolic processes (GO:0009056 line 24 Table 1), proteolysis (GO:0006508 line 5 Table 1), biopolymer and protein catabolic processes (respectively GO:0043285 line 12 Table 1 and GO:0030163 line 10 Table 1). Interestingly, among the down-regulated transcripts involved in proteolysis, PSMB1 (β1 proteasome subunit, line 15 in Table 2), PSMB9 (β1i proteasome subunit, line 17 in Table 2) and PSMB10 (β2i proteasome subunit, line 10 in Table 2), were found to be significantly down-regulated after SMILE silencing.

**Table 1:** Function enrichment of down-regulated transcripts in SMILE siRNA-transfected cells.

	GO NUMBER	GO CATEGORY	TOTAL GENES	CHANGED GENES	p-value
1	GO:0032940	secretion by cell	136	14	0.000550
2	GO:0019752	carboxylic acid metabolic process	303	22	0.002199
3	GO:0006082	organic acid metabolic process	305	22	0.002388
4	GO:0045045	secretory pathway	114	11	0.003544
5	GO:0006508	proteolysis	378	25	0.003965
6	GO:0016192	vesicle-mediated transport	334	22	0.007058
7	GO:0046903	secretion	182	14	0.008350
8	GO:0044255	cellular lipid metabolic process	345	22	0.010152
9	GO:0044260	cellular macromolecule metabolic process	1958	91	0.010267
10	GO:0030163	protein catabolic process	168	13	0.010346
11	GO:0006066	alcohol metabolic process	190	14	0.011947
12	GO:0043285	biopolymer catabolic process	234	16	0.014630
13	GO:0019538	protein metabolic process	2039	93	0.015656
14	GO:0032787	monocarboxylic acid metabolic process	141	11	0.016685
15	GO:0006629	lipid metabolic process	407	24	0.018131
16	GO:0044267	cellular protein metabolic process	1906	87	0.019677
17	GO:0006753	nucleoside phosphate metabolic process	126	10	0.019712
18	GO:0009117	nucleotide metabolic process	126	10	0.019712
19	GO:0044262	cellular carbohydrate metabolic process	189	13	0.025232
20	GO:0055086	nucleobase nucleoside and nucleotide metabolic process	138	10	0.034312
21	GO:0006807	nitrogen compound metabolic process	226	14	0.044629
22	GO:0009308	amine metabolic process	206	13	0.045829
23	GO:0009057	macromolecule catabolic process	291	17	0.046136
24	GO:0009056	catabolic process	448	24	0.048405

**Table 2:** List of the genes involved in proteolysis function (GO:0006508).

	GENE NAME	TOTAL GENES	CHANGED GENES	p-value	FOLD CHANGE
1	CASP9	378	25	0.00396506	0.272137
2	SCRN1	378	25	0.00396506	0.825240
3	LONRF1	378	25	0.00396506	0.650918
4	ABHD4	378	25	0.00396506	0.553770
5	MMP9	378	25	0.00396506	0.813543
6	CRADD	378	25	0.00396506	0.584922
7	YME1L1	378	25	0.00396506	0.702569
8	SRGN	378	25	0.00396506	0.535910
9	NLN	378	25	0.00396506	0.706486
10	PSMB10	378	25	0.00396506	0.603119
11	UBE2N	378	25	0.00396506	0.587638
12	LAP3	378	25	0.00396506	0.834631
13	C1S	378	25	0.00396506	0.489116
14	CTSC	378	25	0.00396506	0.451692
15	PSMB1	378	25	0.00396506	0.756877
16	PCSK1	378	25	0.00396506	0.347323
17	PSMB9	378	25	0.00396506	0.302844
18	RNF11	378	25	0.00396506	0.413019
19	USP48	378	25	0.00396506	0.651608
20	FBXO21	378	25	0.00396506	0.441026
21	USP40	378	25	0.00396506	0.767610
22	UBE2H	378	25	0.00396506	0.576337
23	CPA4	378	25	0.00396506	0.690188
24	USP18	378	25	0.00396506	0.555519
25	OMA1	378	25	0.00396506	0.539055

**Figure 3:**
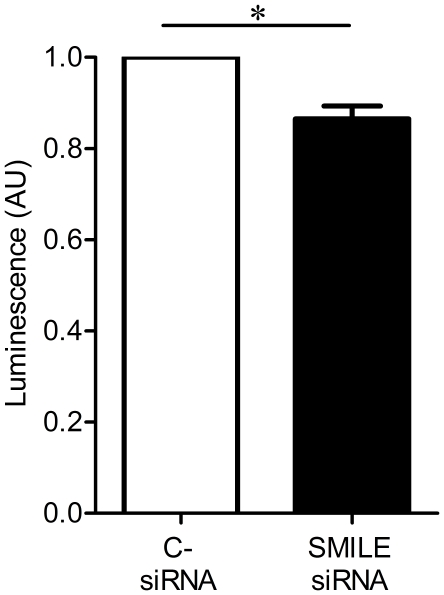
Chymotrypsin-like activity of the proteasome in SMILE siRNA-transfected Hela cells. The luminescent signal measured in arbitrary units (AU) is proportional to the amount of proteasome activity and is decreased in SMILE siRNA versus control (C- siRNA) siRNA-transfected Hela cells (*p = 0.0313, Wilcoxon signed rank test).

Because SMILE transcript down-regulation decreases transcripts involved in protein degradation, we tested whether SMILE was involved in proteolysis. We measured the chymotrypsin-like activity of the proteasome in both SMILE siRNA and control siRNA-transfected HeLa cells. SMILE siRNA-transfected HeLa cells displayed a significantly decreased chymotrypsin-like activity compared to control siRNA-transfected cells (Figure 3, *p = 0.0313, Wilcoxon signed rank test). The findings of SMILE interaction with PDIA3 in the endoplasmic reticulum, together with SMILE modulation of transcripts involved in protein catabolism and chymotrypsin-like activity of the proteasome, suggest that SMILE may play a role in the control of proteolysis *via* proteasome activity in the endoplasmic reticulum.

### SMILE silencing does not affect cell growth but sensitizes HeLa cells to ER stress

**Figure 4:**
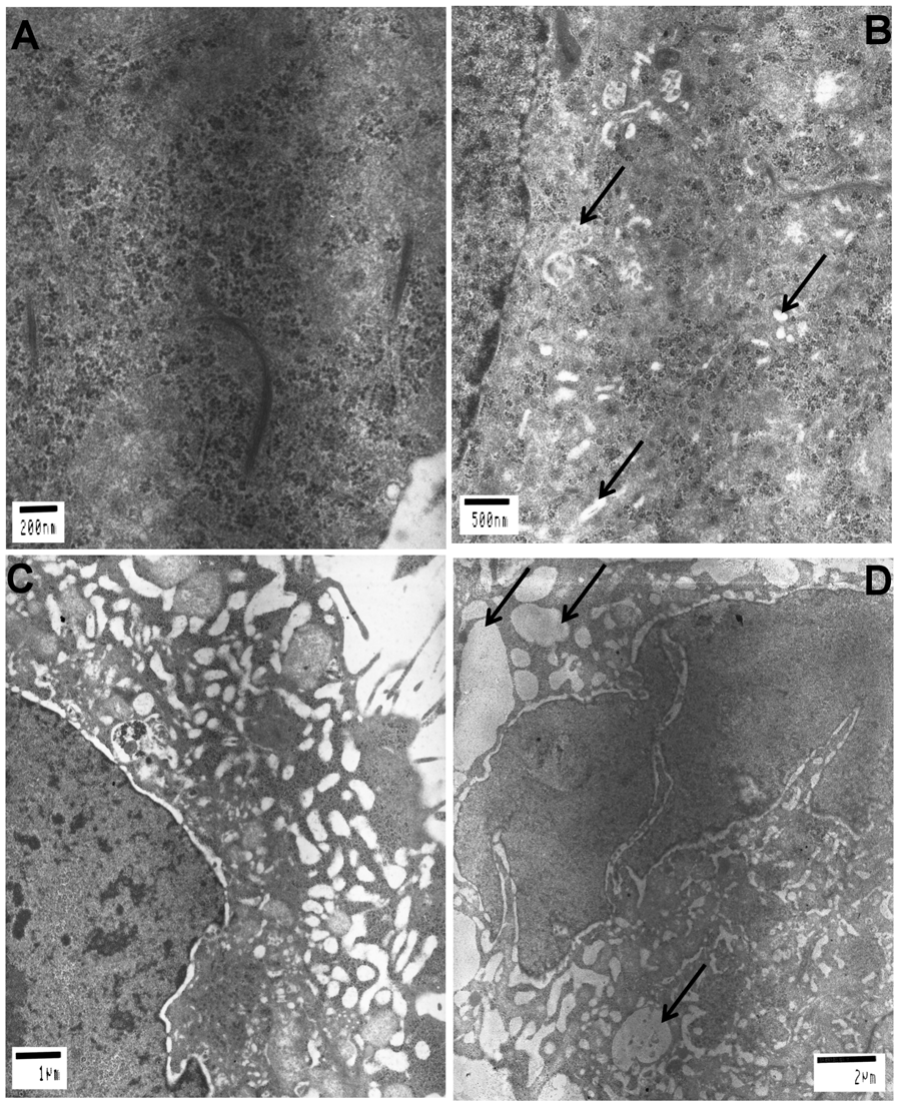
Endoplasmic reticulum hypertrophy in SMILE siRNA-transfected HeLa cells. Control (A) and SMILE (B) siRNA transfected HeLa cells cultured 24 h with RPMI+vehicle (DMSO). Endoplasmic reticulum vacuolization in HeLa cells treated 24 h with 20 nM Bortezomib and transfected with SMILE siRNA (D) compared to cells transfected with control siRNA (C).

To more precisely study the effects of SMILE siRNA on cell morphology, we performed electronic microscopy (EM) analysis in SMILE siRNA and control siRNA-transfected cells. At an ultra structural level, resting control siRNA-transfected cells displayed a well-conserved overall architecture and organization. In contrast, SMILE down-regulation induced ER hypertrophy associated with a reduction of free ribosomes as compared to control cells (Fig. 4A and B), suggesting that down-regulation of SMILE affects ER function. Improperly folded protein degradation is a main actor of ER stress *via* accumulation in the ER lumen. We thus hypothesized that down-regulation of SMILE would sensitize cells to the effect of Bortezomib (a 26S proteasome inhibitor inducing ER stress). To address this question, we performed EM analysis in SMILE siRNA and control siRNA transfected HeLa cells treated with Bortezomib (20 nM for 24 h). As expected, Bortezomib treatment induced ER hypertrophy in control cells (Figure 4C). SMILE siRNA-transfected cells displayed an increased sensitivity to Bortezomib with dramatic ER enlargement and vacuolization and features of cellular disorganization and injury (Figure 4D). These results suggest that SMILE down-regulation sensitizes cells to ER stress.

### The down-regulation of SMILE/TMTC3 increases ER stress and impairs long-term cell survival

**Figure 5:**
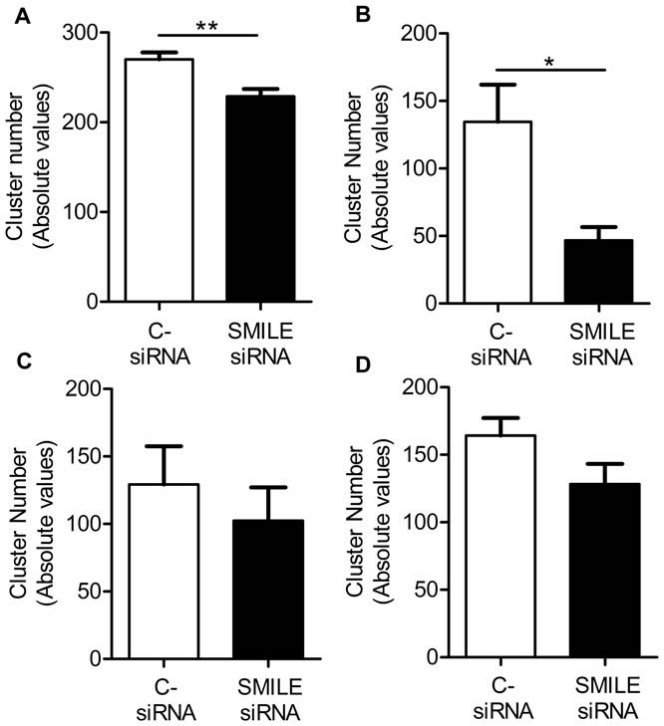
Transfected HeLa cell behavior when undergoing endoplasmic reticulum stress. Graphic representations of viable cell cluster numbers in clonogenic assays. (A) Absolute number of clusters of cells transfected with control siRNA (white bars) compared to cells transfected with SMILE siRNA (black bars) (**p = 0.0045, Mann-Whitney test). Absolute number of clusters of cells transfected with control siRNA (white bars) compared to cells transfected with SMILE siRNA (black bars) and treated with 5 nM Bortezomib (B, *p = 0.0317, Mann-Whitney test), 100 nM Thapsigargin (C, *p* = 0.3939, Mann-Whitney test) or 180 nM Etoposide (D, *p* = 0.4, Mann-Whitney test).

To further determine if SMILE siRNA-mediated down-regulation sensitizes HeLa cells to ER stress and if this is mediated by proteasome activity, we monitored the effects of different drugs inducing various stresses on HeLa cells after SMILE silencing in long-term cultures (7 days). Besides Bortezomib, we used Thapsigargin, a blocker of sarco/endoplasmic reticulum Ca^2+^/ATPase, which induces proteasome-independent ER toxicity. Moreover, Etoposide, an inhibitor of topoisomerase II, that induces cytotoxicity in an ER-independent manner, was also used as a negative control. We compared the effects of a seven-day, dose-response treatment with these drugs in HeLa cells transfected with either SMILE siRNA or control siRNA in clonogenic survival assays. As illustrated in Figure 5A, without any treatment, HeLa cells transfected with SMILE siRNA displayed a decreased number of cell clusters compared to cells transfected with control siRNA (**p = 0.0045, Mann-Whitney test). Bortezomib, Thapsigargin and Etoposide induced a dose-dependent decrease in the cluster numbers in both cells transfected with control or SMILE siRNA, showing that these drugs are effective (Significance of *p* = 0.0001 for the dose-effects of Bortezomib, Thapsigargin and Etoposide, Two-way ANOVA, *data not shown*) We observed that a large dose of Bortezomib induced a significantly greater decrease in the number of clusters constituted by SMILE siRNA-transfected cells compared to control siRNA-transfected cells. These data confirmed the electronic microscopy and suggested that cells lacking SMILE are more sensitive to the toxic effect of an ER stressor that blocks proteasome activity than control siRNA-transfected cells (Figure 5B, *p = 0.0317, Mann-Whitney test). Compared to Bortezomib effects, control and SMILE siRNA-transfected cells treated with Thapsigargin or Etoposide displayed the same decrease in the number of clusters, indicating a similar toxicity of these two drugs on cells lacking SMILE mRNA (Figure 5C and 5D). These results suggest that HeLa cells lacking SMILE mRNA are more sensitive to ER stress dependent on proteasome activity blockade compared to other stresses.

### Down-regulation of SMILE/TMTC3 induces upregulation of XBP-1 transcription

**Figure 6:**
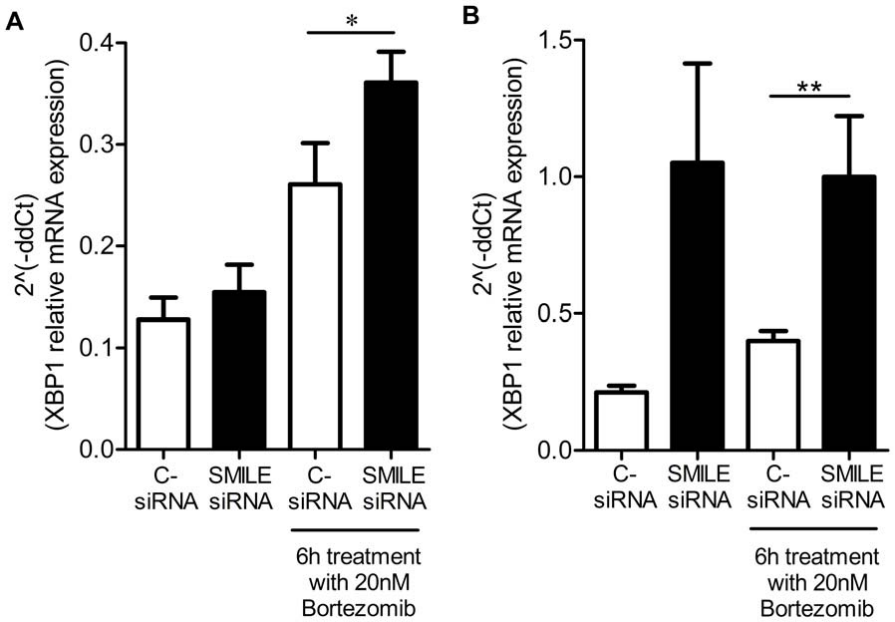
XBP-1 mRNA expression in control versus SMILE siRNA-transfected cells treated or not with Bortezomib 20 nM. (A) XBP-1 transcripts are significantly increased in SMILE siRNA-transfected HeLa cells after 6 h treatment with Bortezomib at 20 nM (*p = 0.0156, Wilcoxon signed rank test). (B) XBP-1 transcripts are also significantly increased in SMILE siRNA-transfected keratinocytes after 6 h treatment with Bortezomib at 20 nM (**p = 0.0078, Wilcoxon signed rank test), and are increased in SMILE siRNA-transfected keratinocytes without treatment (*p* = 0.0547, Wilcoxon signed rank test).

In order to determine whether there is a direct link between SMILE down-regulation and ER stress, we further tested XBP-1 expression in HeLa cells transfected with SMILE siRNA and treated 6 h with 20 nM Bortezomib. XBP-1 is a stress response protein activated upon exposure to ER stress and allowing transcription of genes of the Unfolded Protein Response. SMILE mRNA down-regulation resulted in significant XBP-1 transcript overexpression after Bortezomib treatment (Figure 6A, *p = 0,0156, Wilcoxon signed rank test). This experiment was confirmed on primary cells (human keratinocytes). SMILE mRNA expression was checked by RT-PCR and decreased by almost 70% in resting keratinocytes transfected with SMILE siRNA as compared to cells transfected with Stealth RNAi negative control Low GC (*p = 0.0418, Wilcoxon signed rank test, mean replicate values of four independent experiments, *data not shown*). As shown in figure 6B, SMILE transcript silencing and 6 h-Bortezomib treatment also induced a significant increase in XBP-1 transcription (**p = 0.0078, Wilcoxon signed rank test). Interestingly, SMILE transcript silencing without proteasome blockade also induced an increase in XBP-1 transcription in keratinocytes (*p* = 0.0547, Wilcoxon signed rank test), suggesting that epithelial primary cells are more susceptible to SMILE transcript silencing alone and that SMILE transcript modulation directly impacts ER stress responses.

## Discussion

Although immunological tolerance has been achieved in animal models, its translation into the clinic has not yet been feasible and remains highly experimental in both non human primates and humans. Nevertheless, compelling evidence has accumulated showing that some transplant recipients permanently accept their kidney or liver grafts in the absence of immunosuppressive therapy [5], [10], [11], [12]. Along these lines, during the last decade, significant efforts have been made among the transplant community (*Reprogramming the Immune System for Establishment of Tolerance* and *Indices of Tolerance*) in Europe [11] and (*Immune Tolerance Network*) in the US [12] to identify biological signatures of “operational tolerance”.

We previously identified a list of 49 genes which were able to discriminate operationally tolerant patients from other cohorts of transplant patients [6]. SMILE/TMTC3 was one of the genes found to be differentially expressed in the blood from operationally tolerant patients compared to stable and rejecting patients and whose function was unknown. Confirming the latter study, a differential expression of SMILE transcripts was additionally reported by the team of Newell et al. between a cohort of 25 operationally tolerant patients and stable patients (data available on Gene Expression Omnibus Datasets under reference GSE22229) [12]. The modulation of SMILE transcripts in the blood of operationally tolerant patients and patients with chronic-antibody mediated rejection patients and the independence of SMILE transcript levels to external confounding factors suggest that SMILE may have a potential implication in controlling graft status. However, as there is no described cellular or clinical role for SMILE, it is not yet known if SMILE has an active role in the establishment of tolerance, or if this molecule is a passive biomarker of tolerance. Thus, the present study was conducted to further explore the potential functions of SMILE. We report that SMILE interacts with PDIA3, which has a crucial role in glycoprotein folding in endoplasmic reticulum [22], in the loading of peptide on MHC class I in endoplasmic reticulum [13] and which is overexpressed during ER stress. The interaction between SMILE and PDIA3 was initially identified in a yeast Two-Hybrid screen and confirmed by immunohistochemistry showing an endoplasmic reticulum colocalization of the two molecules. We also showed here that siRNA-mediated SMILE knock-down in HeLa cells induces a decrease in several types of transcripts involved in protein catabolism and proteolysis. Among these transcripts we found that several immunoproteasome subunits (PSMB1, PSMB9 and PSMB10) were modulated, suggesting that SMILE exerts its function *via* the proteasome pathway. As expected, proteasome activity assessed by chymotrypsin-like activity was decreased in SMILE siRNA-transfected cells as compared to control siRNA-transfected cells. These results suggest that SMILE might have a role in protein folding and/or degradation, exerting its function *via* the proteasome pathway.

Incorrect folding of proteins in cells is counteracted by the Unfolded Protein Response (UPR). If UPR is not sufficient to process protein overload in the ER, this pathway can be deleterious and lead to cell apoptosis or autophagy [14], [15]. To assess the involvement of SMILE in ER stress responses and protein catabolism, we treated SMILE siRNA-transfected cells with various stressors, including Bortezomib, a proteasome inhibitor. SMILE down-regulation and/or Bortezomib treatment induced dramatic ER enlargement and features of cellular injury. Furthermore, Bortezomib inhibition of long-term cellular growth was strongly enhanced in SMILE siRNA-transfected cells. Interestingly, the toxicity of Thapsigargin, an ER stressor whose effects are unrelated to proteasome inhibition, was independent of the level of SMILE expression on the cell response to stress. Thus, SMILE transcript inhibition increased sensitivity to ER stress dependent on protein overload induced by the proteasome inhibitor Bortezomib. One arm of the UPR response involves the spliced transcript XBP-1. In this study, we showed that SMILE silencing directly increased XBP-1 transcript expression after 6 hours of Bortezomib treatment. Altogether these data suggest that in HeLa cells, proteasome pharmacological inhibition and SMILE silencing act in a synergistic way, likely by blocking protein degradation or modification for degradation. As suggested in the literature, blockade of protein degradation induces accumulation of misfolded proteins in the ER and leads to ER stress, and thus to XBP-1 overexpression [16].

Interestingly, a recent study by Fasanaro *et al.* reported that SMILE/TMTC3 mRNA is inversely modulated after miR-210 over-expression or inhibition [17]. Of note, miR-210 expression is induced by hypoxia, which was shown to induce UPR as a pro-survival mechanism in tumor cells [18]. One of the responses to hypoxia via miR-210 involves indirect targets implicated in amino acid catabolism [17]. Our results in proteolysis suggests that SMILE may be part of the response to hypoxia - and thus to ER stress - via miR-210 or not. Moreover, our DNA chip analysis revealed that SMILE down-regulation in HeLa cells affects the secretory pathway as well as vesicle-mediated transport (GO:0045045 and GO:0016192). Interestingly, membrane trafficking is one of the functions that is modified in response to miR-210 modulation and that could be set off by hypoxia, according to Fasanaro *et al.* [17]. Thus, this work supports our results for SMILE having a role in proteolysis and being potentially an actor of the ER stress response. Regarding the fact that SMILE was discovered in PBMCs of patients, it may play a direct role in the immune cell physiology in long-term graft function. The role of the UPR, and particularly of XBP-1, in the mammalian immune system [19], [20] and in inflammation has been clearly demonstrated [21]. Indeed, the stress response is involved in a variety of immune cells such as dendritic cells [20], [22], macrophages [23] or B cells [24], [25], [26] and depend on the UPR and notably XBP-1 for their development and/or function. This could be of potential interest given the recent studies showing that operationally tolerant patients display a particular B cell profile highlighting a possible abnormal B cell differentiation process in these patients [11], [12], [27], [28]. A recent paper have reported that the STAT3/IL-6 pathway, that has also been shown to be involved in ER stress [29], [30], is activated neither in operationally tolerant patients nor in rejecting patients [31]. These results that do not confort our hypothesis may be due to the fact that the STAT3/IL-6 pathway is not the only signaling pathway reflecting UPR activity, and the absence of its activity in operationally tolerant or rejecting patients may not preclude the absence of UPR activity in the PBMCs of these patients. Growing evidence suggests that the selectivity of Bortezomib for myeloma cells may be explained by an increased susceptibility of myeloma cells to ER stress-induced apoptosis [32]. In addition, Bortezomib is not only selective for cancerous cells, as recent studies showed that primary B cells, that are largely dependent on UPR and proteasome activity to produce antibodies, are sensitive to Bortezomib. This treatment was shown to decrease donor-specific antibodies in renal transplant patients in recent studies [33], [34]. Our results showed that primary cells are far more sensitive to SMILE transcript silencing than HeLa cells, as there was no need for Bortezomib treatment to induce XBP-1 overexpression in SMILE-silenced keratinocytes. These results suggest that SMILE transcript modulation in immune cells may have an impact on the function of the cell and particularly on its response to ER stress. They allow a function in ER stress response to be attributed to this molecule, which was previously unknown. Moreover, it opens up new perspectives about ER stress and graft immune regulation, given the role of the ER stress response in immune cells. SMILE may have a potential role in these cell types related to the emerging role of the ER stress response in transplantation. We also envisage a role for SMILE in the graft itself, in addition to recent works showing ER stress emerging as an actor at the graft level [35], [36], [37].

To conclude, further studies are needed to analyze the effects of SMILE transcript modulation in immune cells. This molecule and its link with endoplasmic reticulum stress could be of potential relevance in the field of organ transplantation.

## Materials and Methods

### Patients

The study was performed on 42 blood samples. All patients and healthy volunteers (HV) who participated in this study signed an informed consent and the study was approved by the University Hospital Ethical Committee (Nantes, France). The clinical parameters of these patients are described in detail in Table S1.

- Patients under standard immunosuppressive therapy with stable graft function (STA; n = 9; patients with Cockroft creatinine clearance >40 mL/min and proteinuria <1 g/24 h) for at least 3 years with donor-specific antibodies for 2 out of 9 patients. No biopsies were available for these patients because they presented no deterioration of graft function (certain cDNA samples were prepared by TcLand Expression S.A., Nantes, France). These patients were under anti-metabolites (mycophenolate mofetil or azathioprine), calcineurin inhibitors (Cyclosporin A or FK506) and/or steroids.
- Operationally tolerant patients: patients with stable graft function (TOL; n = 8; Cockroft creatinine clearance >40 mL/min and proteinuria <1 g/24 h) for at least 1 year (median 12.5 years, range 5–30 years) without immunosuppressive treatment. Immunosuppressive treatment was stopped due to non compliance (n = 6), post-transplant lymphoproliferative disorder (n = 1) or calcineurin inhibitor toxicity (n = 1). No biopsies were available for these patients since biopsy was refused by our Centre's Ethical Committee.
- Patients with chronic antibody mediated rejection: Patients under standard immunosuppressive therapy with biopsy-proven chronic antibody-mediated rejection (transplant glomerulopathy, positive for C4d and anti-donor HLA antibodies) (CAMR; n = 14) according to the updated Banff classification criteria [38]. Chronic AMR was diagnosed on biopsies performed in the context of a progressive deterioration of renal function (Cockroft creatinine clearance <40 mL/min and/or proteinuria >1 g/24 h).

### Peripheral Blood Mononuclear Cells

Peripheral blood from healthy volunteers and patients was collected in EDTA Vacutainers, and PBMC were separated by density centrifugation using Lymphosep, lymphocyte separation media (Bio West, Nuaille, France). PBMC were stored in TRIzol (Invitrogen, Cergy Pontoise, France) at −80°C until use.

### RNA Extraction and Preparation of cDNA

RNA was extracted from human PBMC, HeLa cells and keratinocytes using the TRIzol method (Invitrogen) according to the manufacturer's instructions. Genomic DNA was removed by DNase treatment (Roche, Indianapolis, IN). RNA concentration was calculated using a Nanodrop ND1000 spectrophotometer (NanoDrop Technologies, Wilmington, DE). RNA was reverse transcribed into cDNA using polydT oligonucleotide and Maloney leukemia virus reverse transcription (Invitrogen).

### Real-Time Quantitative PCR

Real-time quantitative PCR was performed in an Applied Biosystems GenAmp 7700 or 7900 sequence detection system (Applied Biosystems, Foster City, CA) using a commercially available primer and probe set for human SMILE/TMTC3 (Applied Biosystems; Hs00699202_m1) and XBP-1 (Applied Biosystems; Hs00231936_m1). The housekeeping gene hypoxanthine phosphoribosyl transferase (HPRT, Applied Biosystems; Hs99999909_m1) was used as an endogenous control to normalize RNA starting quantity. Relative expression between a given sample and a reference sample was calculated according to the 2^−ddCt^ method after normalization to HPRT with results expressed in arbitrary units.

### Culture and treatment of Human cervical cancer cell line (HeLa) and keratinocytes

Human cervical cancer cells HeLa were cultured in RPMI 1640 medium (Invitrogen, Cergy Pontoise, France) supplemented with 10% fetal bovine serum, 1% penicillin-streptomycin, 1% glutamine, 1% Hepes, 1% non-essential amino acids and 1% sodium pyruvate. Keratinocytes were kindly provided by Dr. Halary (INSERM UMR 643, Nantes, France) and cultured in Keratinocyte Growth Medium (#C-20011, PromoCell, Heidelberg, Germany). SMILE knockdown was achieved with specific small interfering RNA (TMTC3 Stealth RNAi™ siRNA, # HSS136195), Lipofectamine™ RNAiMAX for HeLa cells and Lipofectamine™ 2000 transfection reagent+OptiMEM for keratinocytes, purchased from Invitrogen. Cells were transfected using the manufacturer's protocol.

### Yeast two-hybrid screen

Yeast two-hybrid screening was performed by Hybrigenics Services SAS, France (http://www.hybrigenics-services.com). The coding sequence for aa 1–230 of PDIA3 (GenBank accession number gi: 67083697) was PCR-amplified and cloned into pB28 as a C-terminal fusion to LexA (N-LexA-PDIA3-C). The construct was checked by sequencing the entire insert and used as a bait to screen a random-primed human brown adipocyte cDNA library constructed into pP6. pB28 and pP6 derive from the original pBTM116 [39] and pGADGH [40] plasmids, respectively. 150 million clones (15-fold the complexity of the library) were screened using a mating approach with Y187 (matα) and L40ΔGal4 (mata) yeast strains as previously described [41] and positive clones were selected on a medium lacking tryptophan, leucine and histidine, and supplemented with 0.5 mM 3-aminotriazole to handle bait autoactivation. The prey fragments of the positive clones were amplified by PCR and sequenced at their 5′ and 3′ junctions. The resulting sequences were used to identify the corresponding interacting proteins in the GenBank database (NCBI) using a fully automated procedure. A confidence score (PBS, for Predicted Biological Score) was attributed to each interaction as previously described [8].

### Preparation of odontoblast culture

Dental pulps were obtained from healthy human third molar germs (from 14- to 16-year-olds) extracted for orthodontic reasons with the informed consent of the participants and their parents, in accordance with the French Public Health Code and following a protocol approved by the local ethics committee. Pulps were processed for cultured odontoblast-like cells as described previously [42] and treated during 24 hours with Bortezomib 20 nM (Millennium Pharmaceuticals, Inc, Cambridge, United Kingdom).

### Immunohistochemistry

Odontoblast cell cultures were fixed in 4% paraformaldehyde-0,025% saponin-PBS for 30 min at 4°C, then rinsed in PBS-0,025% saponin-2 mg/ml bovine serum albumin-0,1 M lysine HCl at 4°C. Intracellular detection of proteins was promoted by the permeabilizing effect of saponin. Cultures were then reacted for double staining with anti-PDIA3 (# HPA003230, Sigma-Aldrich, France) and anti-smile (# ab81473, Abcam, France) antibodies. Subsequently, the cultures were rinsed, incubated with goat anti-mouse Alexa Fluor 594 and goat anti-rabbit 488 (Molecular Probes, Eugene, OR, USA). Observations were made by scanning laser confocal microscopy (Zeiss LSM510, Zeiss, Le Pecq, France) with 40×/1.3 oil immersion objectives. PDIA3 was assigned red, and Smile was assigned green with the laser scanning software. Negative controls were carried out by omission of the primary antibodies or by incubation with normal mouse or rabbit IgG (*data not shown*). Figures from the Z-stack were processed in Adobe Photoshop 6.0 (Adobe Systems,San Jose, CA, USA).

### Transmission Electron Microscopy on transfected and drug-treated Hela cells

SMILE and control siRNA transfected HeLa cells at the 3rd day of culture were fixed in cacodylate buffered 4% glutaraldehyde for 1 h at 4°C, washed in buffer and post-fixed in cacodylate buffered 2% osmium tetroxide for 1 h at room temperature. Cells were dehydrated in increasing concentrations (from 50° to 100°) of ethanol and embedded in Epon. Sections (70 nm-thick) were cut with an Ultracut E ultramicrotome (Leica Microsystems GmbH, Wetzlar, Germany), mounted on copper grids, stained with the Reynolds method and observed on a JEM 1010 electron microscope (Jeol LTD, Tokyo, Japan) at a voltage of 80 kV.

### Gene expression analysis in HeLa cells using DNA chips

RNA samples representing two independent experiments from HeLa cells transfected 24 hours with negative control or SMILE siRNA and activated or not with 20 µM PMA (Phorbol 12-myristate 13-acetate) for 6 hours were submitted for analysis. After checking RNA quality, 500 µg of total RNA for each sample were prepared with the Agilent Quick Amp Labeling Kit following the one-color manufacturer's protocol. Each sample was hybridized to a whole human genome microarray (4×44 K Agilent) following the manufacturer's instructions. After scanning, data were extracted with Feature Extraction (Agilent Technologies) were normalized (lowess function in R [43]) and then, negative control spots and background signal were removed. Significance Analysis of Microarrays (SAM) [44] was applied to identify transcripts differentially expressed between SMILE siRNA and control siRNA-transfected cells. For each analysis, we arbitrarily fixed the false discovery rate (FDR) at less than 0.5%. To assess the biological significance of the differentially expressed genes identified with SAM, GOminer software [44], [45] was used to identify the over-represented GO ontology (GO) categories. Only GO categories among the biological process ontology (GO:0008150) were analyzed, and we selected GO categories with enrichment *p*-values inferior to 0.05, and categories with at least 10 genes. All microarray data is MIAME compliant and the raw data has been deposited in a MIAME compliant database, the Gene Expression Omnibus Datasets. The complete list of the probes used and expression analysis has been submitted to Gene Expression Omnibus GEO # GSE21886.

### Proteasome-Glo™ Cell-Based Assay

HeLa cells were seeded in 6-well plates at a density of 8×10^5^ cells per well for 24 h and transfected for 48 h with control and SMILE siRNA as described above. The chymotrypsin-like activity of transfected cells was then assayed with the Proteasome-Glo™ Cell-Based Reagent (Promega, Charbonnières Les Bains, France) according to manufacturer's protocol. Luminescence was read with a VICTOR™ X Multilabel Plate Reader (Perkinelmer, Massachusetts, USA).

### Clonogenic survival assays

Control and SMILE siRNA transfected HeLa cells were seeded in 6-well plates at a density of 500 cells per well and exposed to increasing concentrations of Bortezomib (1.25 nM, 2.5 nM or 5 nM from a 0.1 mg/ml start solution, Millennium Pharmaceuticals, Inc, Cambridge), Thapsigargin (25 nM, 50 nM, 100 nM from a 1 mM start solution, Sigma-Aldrich) or Etoposide (90 nM, 120 Nm, 180 nM from a 50 mM start solution, Sigma-Aldrich) for 24 hours. Controls were performed with vehicle only: H_2_O for Bortezomib and DMSO for Thapsigargin and Etoposide. Then, the drug/medium was removed and cells were allowed to incubate in fresh medium under normal conditions for 7 days. After incubation, cells were fixed with 10% methanol–10% acetic acid and stained with a 0.4% solution of crystal violet. Plating efficiencies were determined for each treatment and normalized to untreated cells. Error bars represent SEM.

### Statistical Analyses

The nonparametric Mann-Whitney test, the nonparametric Wilcoxon matched pairs test and the nonparametric Kruskal-Wallis test were performed when appropriate. Values of *p<0.05, **p<0.01 and ***p<0.001 were considered as significant. ROC curve analysis was performed to determine the cutoff point of SMILE mRNA in blood that yielded the highest combined sensitivity and specificity in diagnosing operational tolerance. The statistical method was devoted to the analysis of the diagnostic properties of SMILE, and the theory of ROC (receiver operating characteristic) curves was applied. More information about this method is available in SD Experimental Procedures.

A statistical analysis was also made in order to study the relationship between SMILE mRNA expression in a cohort of 164 stable patients and different clinical factors that could influence the diagnostic power of this biomarker. SMILE distribution was normalized with a logarithmic transformation and SMILE log-values were predicted thanks to a multiple linear regression model.

## Supporting Information

Figure S1 SMILE mRNA profile in renal transplant patients. The quantity of SMILE mRNA transcripts is increased in PBMC of operationally tolerant patients (TOL, n = 8) compared to patients with stable graft function under standard immunosuppressive therapy (STA, n = 164, **p<0.01) and deteriorating graft function under standard immunosuppressive therapy with biopsy-proven chronic antibody-mediated rejection (CAMR, n = 19, *p<0.01) (**p = 0.0052, Kruskal-Wallis test).
(TIF)

Figure S2 ROC curve analysis measuring the ability of SMILE mRNA quantity to correctly distinguish operationally tolerant patients from patients with chronic antibody-mediated rejection.
(TIF)

Figure S3 Analysis of SMILE association with continuous explicative variables in a group of 164 stable kidney transplant recipients.
(TIF)

Figure S4 Analysis of SMILE association with continuous qualitative variables in a group of 164 stable kidney transplant recipients.
(TIF)

Figure S5 Graphic representation of the domain architecture of PDIA3 (A) and SMILE (B). The blue boxes represent the full-length proteins. The pink rectangle shows the bait fragment of PDIA3 which was used for the yeast two-hybrid (Y2H) screen. The orange rectangle represents the smallest interacting domain (SID) of SMILE. The black lines show the seven independent prey fragments of SMILE that were identified in the Y2H screen using PDIA3 as bait. Functional and structural domains are indicated by colored rectangles: yellow, signal peptides; red, transmembrane domains; magenta, coiled-coil domains; grey: predicted functional domains (PFAM database). The numbers indicate the amino acid positions of the corresponding domains.
(TIF)

Figure S6 SMILE transcript level analysis in non-transfected HeLa cells (HeLa alone), control siRNA-transfected HeLa cells (C- siRNA) and SMILE siRNA-transfected HeLa cells (SMILE siRNA) (***p = 0.0002, Mann-Whitney test).
(TIF)

Table S1 Patients included in analysis of PBMCs.
(XLS)
