# Supplementary figures and images for: The Involvement of SMILE/TMTC3 in Endoplasmic Reticulum Stress Response
Source: PLoS One. 2011 May 16;6(5):e19321. doi: 10.1371/journal.pone.0019321 (PMC3095597; doi:10.1371/journal.pone.0019321)

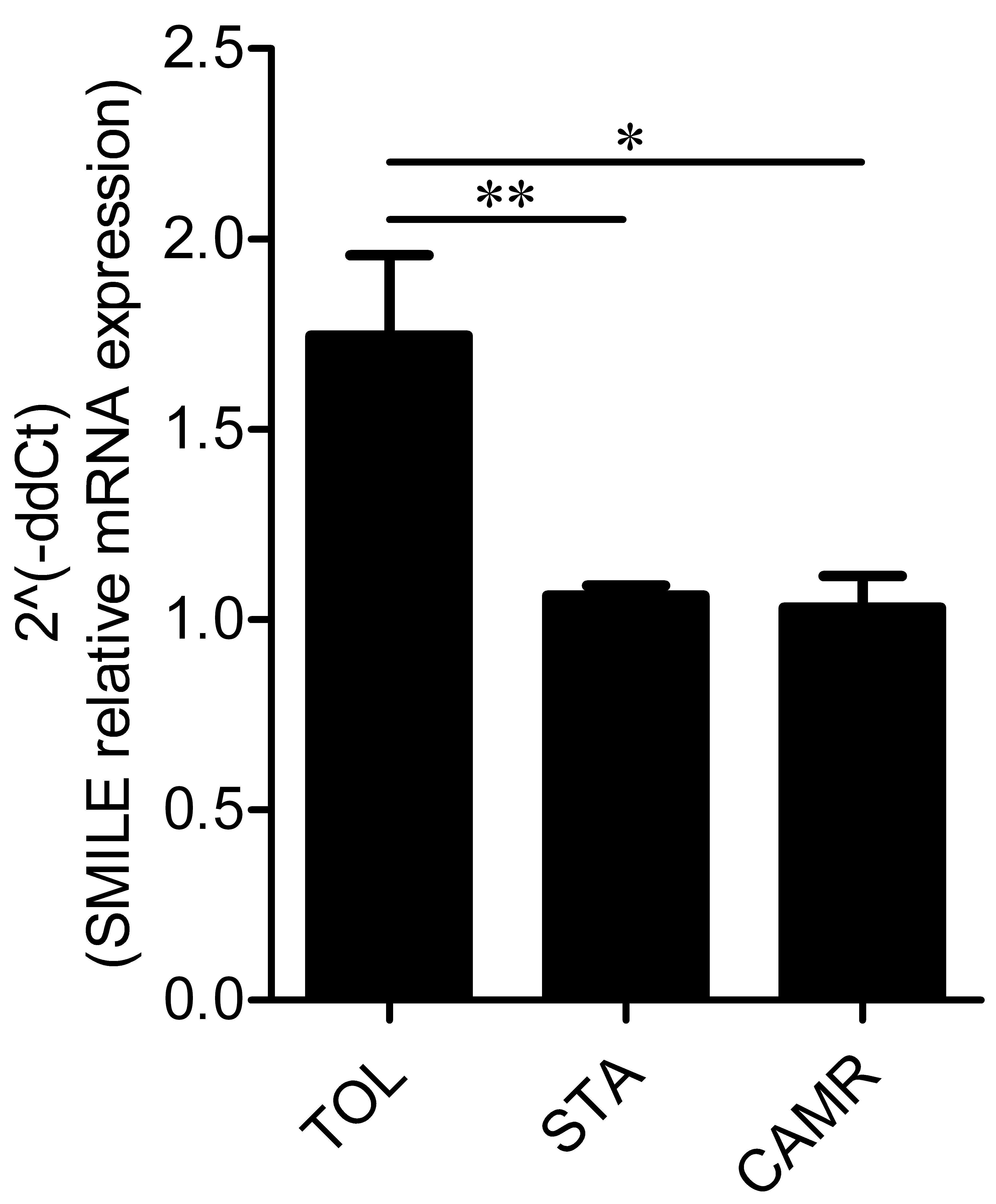

Supplement: Figure S1 — SMILE mRNA profile in renal transplant patients. The quantity of SMILE mRNA transcripts is increased in PBMC of operationally tolerant patients (TOL, n = 8) compared to patients with stable graft function under standard immunosuppressive therapy (STA, n = 164, **p<0.01) and deteriorating graft function under standard immunosuppressive therapy with biopsy-proven chronic antibody-mediated rejection (CAMR, n = 19, *p<0.01) (**p = 0.0052, Kruskal-Wallis test). (TIF) [file pone.0019321.s001.tif]

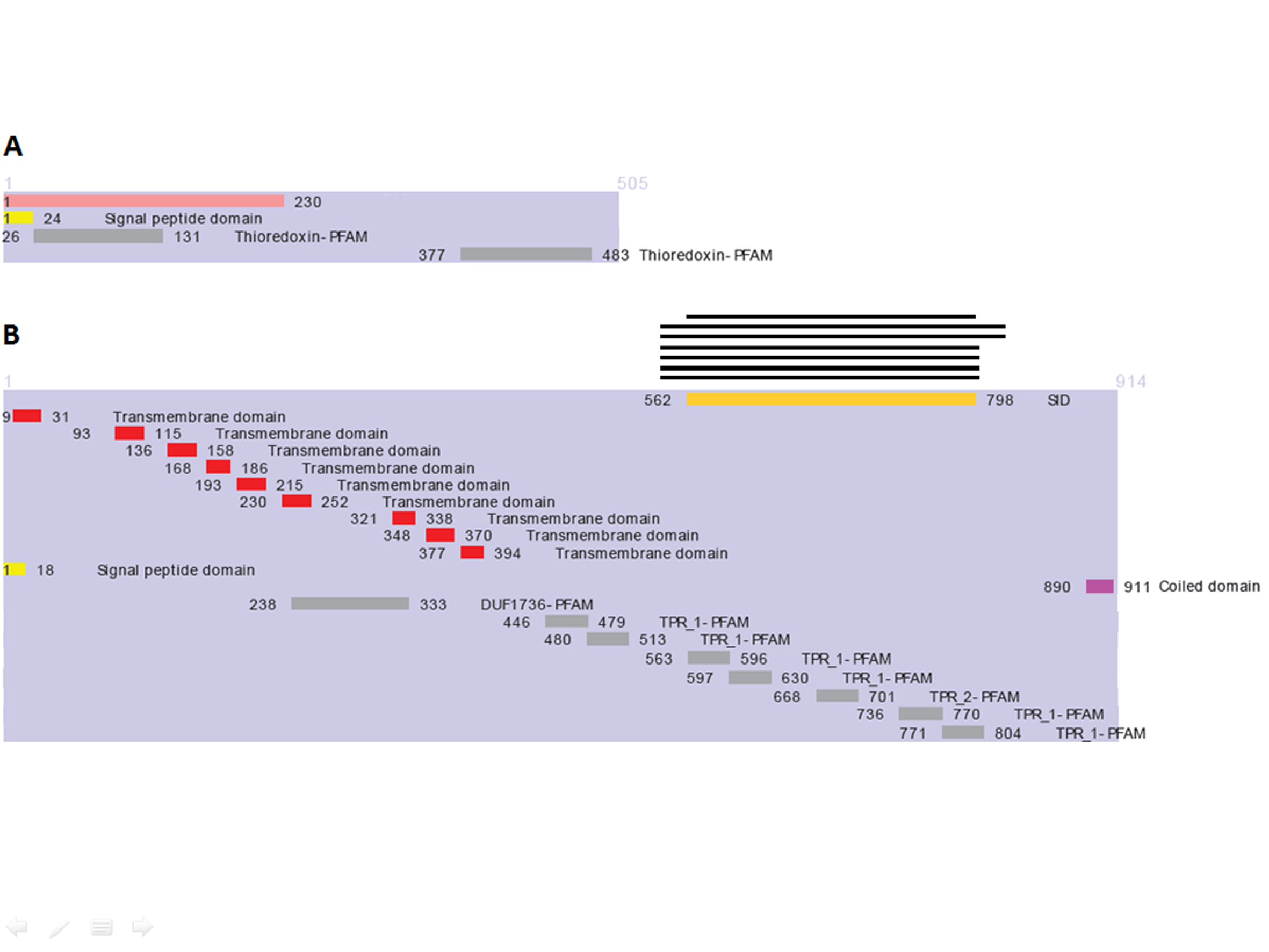

Supplement: Figure S5 — Graphic representation of the domain architecture of PDIA3 (A) and SMILE (B). The blue boxes represent the full-length proteins. The pink rectangle shows the bait fragment of PDIA3 which was used for the yeast two-hybrid (Y2H) screen. The orange rectangle represents the smallest interacting domain (SID) of SMILE. The black lines show the seven independent prey fragments of SMILE that were identified in the Y2H screen using PDIA3 as bait. Functional and structural domains are indicated by colored rectangles: yellow, signal peptides; red, transmembrane domains; magenta, coiled-coil domains; grey: predicted functional domains (PFAM database). The numbers indicate the amino acid positions of the corresponding domains. (TIF) [file pone.0019321.s005.tif]

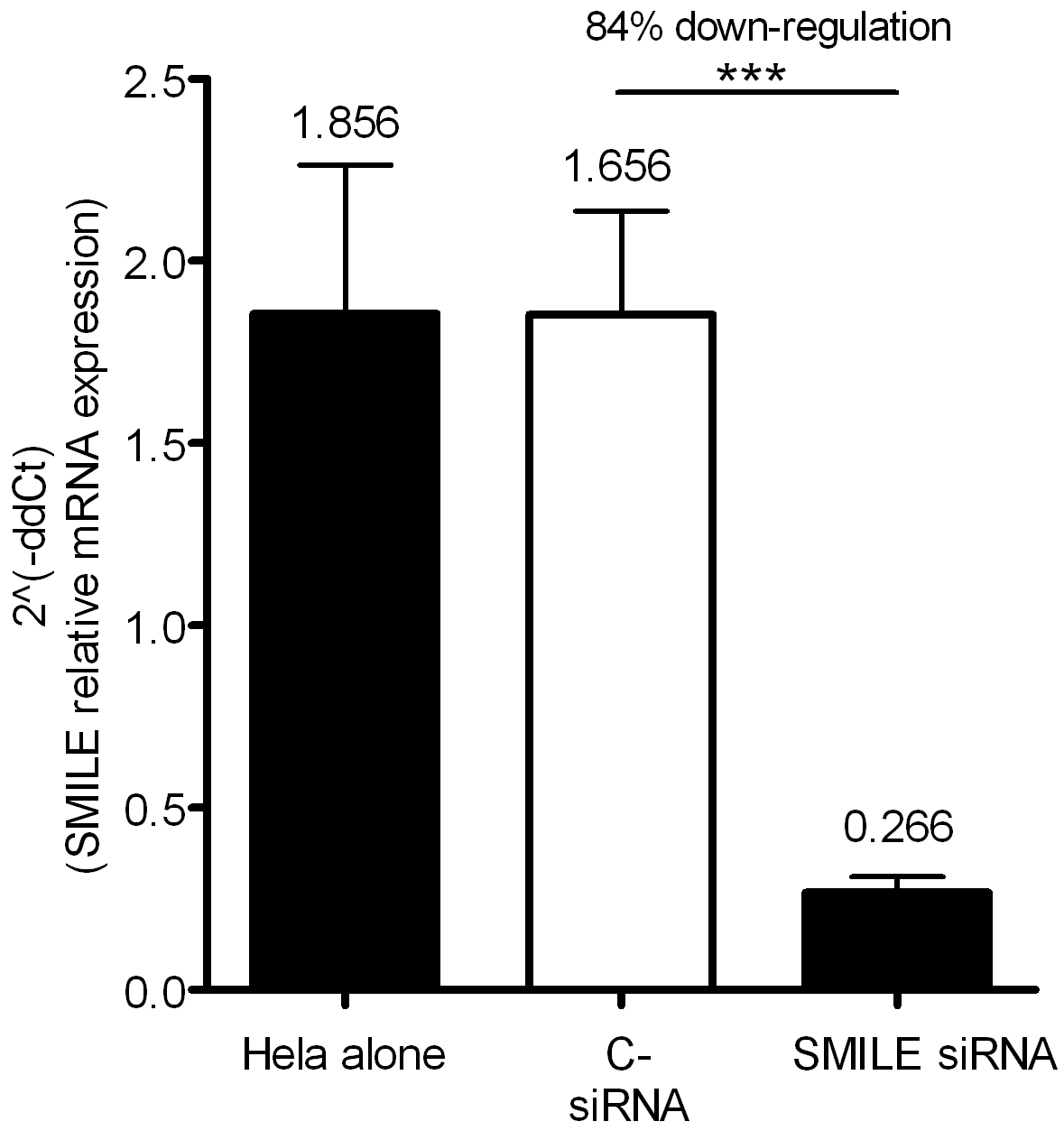

Supplement: Figure S6 — SMILE transcript level analysis in non-transfected HeLa cells (HeLa alone), control siRNA-transfected HeLa cells (C- siRNA) and SMILE siRNA-transfected HeLa cells (SMILE siRNA) (***p = 0.0002, Mann-Whitney test). (TIF) [file pone.0019321.s006.tif]
